# New Trends of Cervical Cancer Incidence in Kazakhstan

**DOI:** 10.31557/APJCP.2021.22.4.1295

**Published:** 2021-04

**Authors:** Nurbek Igissinov, Gulnur Igissinova, Zhansaya Telmanova, Zarina Bilyalova, Dariyana Kulmirzayeva, Zhanar Kozhakhmetova, Saltanat Urazova, Dulat Turebayev, Gaukhar Nurtazinova, Ardak Omarbekov, Aigul Almabayeva, Zhanar Bukeyeva, Dinara Tarzhanova, Altyn Moldabayeva, Marina Zhanaliyeva, Ainagul Kazbekova, Vladimir Openko, Saken Kozhakhmetov, Yerlan Kuandykov

**Affiliations:** 1 *Astana Medical University, Nur-Sultan, Kazakhstan. *; 2 *Central Asian Cancer Institute, Nur-Sultan, Kazakhstan. *; 3 *Eurasian Institute for Cancer Research, Bishkek, Kyrgyzstan. *; 4 *Asfendiyarov Kazakh National Medical University, Almaty, Kazakhstan. *; 5 *National Center for Neurosurgery, Nur-Sultan, Kazakhstan. *; 6 *Khoja Akhmet Yassawi International Kazakh-Turkish University, Turkistan, Kazakhstan. *

**Keywords:** Cervical cancer, incidence, trends, Kazakhstan

## Abstract

**Objective:**

The epidemiological features of the cervical cancer (CC) incidence and its spatial and temporal assessment in Kazakhstan were studied.

**Methods:**

The retrospective study was done for the period 2009-2018. Descriptive and analytical methods of oncoepidemiology were used.

**Results:**

During the study period, 16,441 new cases of CC were registered. The average annual crude and age-standardized incidence rate were 18.6±0.5 and 17.7±0.4 cases per 100,000 population of female, respectively, and their trends tended to increase (Т_up_=+2.3%; R^2^=0.708 and Т_up_=+1.9%; R^2^=0.615, respectively). The analysis of ASIR showed unimodal growth with a peak at 50-54 years – 45.3±1.1 cases per 100,000 population of female. Trends of ASIR decreased up to 30 years (T_down_=−1.8%; R^2^=0.111) and 35-59 years (T_down_=−0.9%; R^2^=0.103), in other age groups the trends increased, and were most pronounced in 40-44 (Т_up_=+4.1%; R^2^=0.878) and 65-69 years (Т_up_=+4.4%; R^2^=0.537). Trends in ASR of СС tended to grow in almost all regions, with higher levels in Mangystau (Т_up_=+4.1%; R^2^=0.482) and Aktobe (Т_up_=+6.3%; R^2^=0.846) regions. The cartograms of ASR per 100,000 population of female were allocated according to the following criteria: low – up to 16.3, average – from 16.3 to 19.2, high – above 19.2. The results of the spatial analysis showed the regions with a higher levels of CC incidence rate per 100,000 population of female: East Kazakhstan (19.8), Aktobe (20.0), Almaty (20.1), Kostanay region (20.9), Atyrau (21.7) regions and Almaty city (22.0).

**Conclusion:**

The study of trends of the cervical cancer incidence has the theoretical and practical value: monitoring and evaluation of screening programmes, which are implemented in the country, and conduction of secondary prevention of cervical pathology. Health authorities should consider the obtained results in the in the organization of anti-cancer activities.

## Introduction

Cervical cancer (CC) is a common oncological disease that occupies the 4th place in the structure of oncopathology in women. Thus, about 604 thousand new cases of CC are registered annually, and 342 thousand females worldwide die from this pathology (Ferlay et al., 2020). In some countries, СС accounts for up to 80% of the total oncological incidence of the female genital organs, although in general, breast cancer is the first place in the world (Curado et al., 2007; Gretsova et al., 2017; Zhang et al., 2020). High standardized incidence rates (age-standardized incident rate) of CC per 100,000 population of female were found in countries such as Zambia (65.5), Malawi (67.9) and Eswatini (84.5) (Ferlay et al., 2020; Curado et al., 2007; Gretsova et al., 2017; Zhang et al., 2020).

Epidemiological studies have long shown a close relationship between the occurrence of СС and sexual behavior. The decisive factors are the number of sexual partners and the early age at first intercourse (Ngoma and Autier, 2019; Zhang et al., 2020).

The viral hypothesis takes the leading position in the theory of CC origin, in which the leading role is given to human papillomavirus and indirectly herpessimplexvirus-2 and cytomegalovirus infection. Prolonged exposure to various urogenital infections in women causes immunodeficiency conditions that lead to the risk of pathology of the cervical mucosa (zur Hausen and de Villiers, 1994; Fernandes et al., 2015; Song et al., 2015; Omire et al., 2020; Mac and Moody, 2020).

However, if these causes of the disease are identified when contacting a gynecologist or during preventive examination, serious measures should be taken (Santoso et al., 2010).

Many countries with different preventive screening programmes have seen significant declines in CC mortality over the past half century. Preventive studies of vaginal and cervical smear, PAP tests and HPV DNA tests are highly effective diagnostic tests in accordance with the recommendations of world oncology services. The goal of CC screening is to detect lesions at an early stage before cancer develops.

Recently, in Kazakhstan, the active detection of precancerous and background cervical diseases, as well as the early manifestation of cancer, are undoubtedly associated with mass cytological screening (Kaidarova et al., 2017).

Despite screening, the incidence and mortality of CC does not decrease, but increases (Bray et al., 2018; Ferlay et al., 2019; Fontham et al., 2020). Despite screening, the incidence and mortality of CC increases (Bray et al., 2018; Ferlay et al., 2019; Fontham et al., 2020). But, there are numerous convincing epidemiological data which show that since the introduction of Pap in countries with well organized screening programs, and with wide population coverage, both incidence of, and mortality from cervical cancer has significantly decreased (Safaeian et al., 2007; Comprehensive Cervical Cancer Control, 2014; U.S. Cancer Statistics Working Group, 2020). Some studies on this topic have been carried out in Kazakhstan (Igissinov et al., 2012; Aimagambetova and Azizan, 2018; Balmagambetova et al., 2019). However, the problem remains relevant and interesting from an epidemiological point of view. This paper reviews the CC incidence in Kazakhstan in recent years.

## Materials and Methods

*Cancer registration and patient recruitment*

The population of republic of Kazakhstan as the 2018 census was 18.2 million, of which 9.36 million were females (Bureau of National Statistics, 2018), while the dynamics of the female population increased by 13.1% compared to 2009. The cancer registry of the population of Kazakhstan covers 14 regions and cities of national significance-Almaty and Astana (now the city of Nur-Sultan). New cases of cervical cancer were extracted from the accounting and reporting forms of the Ministry of Health of the Republic of Kazakhstan – form 7 and form 35, which were formed from the register of oncological diseases based on the administrative-territorial division of the republic from 2009 to 2018 using the International Disease Code 10, code C53.

*Population denominators*

Population denominators for calculation of incidence rates were provided by the Bureau of National Statistics for 2009-2018. At the same time, data on the number of female population of the republic, taking into account the studied regions, are used, all data are presented on the official website (www.stat.gov.kz).

*Statistical analysis*

The main method used in the study of incidence was a retrospective study using descriptive and analytical methods of modern oncoepidemiology. Age-standardized incidence rates (ASRs) were calculated for eighteen different age groups (0-4, 5-9, …, 80-84, and 85+) and ten calendar periods from 2009 to 2018 (1-year intervals). ASRs standardized to the world population proposed by World Health Organization (Ahmad et al., 2001) with recommendations from the National Cancer Institute (1976) were estimated for each studied year.

The extensive, crude and age-specific (ASIR) incidence rates are determined according to the generally accepted methodology used in modern sanitary statistics. The annual averages (M, P), mean error (m), Student criterion, 95% confidence interval (95% CI), and average annual upward/downward rates (T%) were calculated. We did not justify the main calculation formulas in this paper, since they are detailed in the methodological recommendations and textbooks on medical and biological statistics (Merkov and Polyakov, 1974; Glanc, 1999; dos Santos Silva, 1999). However, the following are some of them:

**Extensive rate (ER)=(n×100%)/N**                    ** (1)**

where **n** is the number of cases from the general population N.

**Crude rate (CR)=(n×100,000)/N**                    ** (2)**

where **n** is the number of diseases; **N** is the average population.

The dynamics of incidence rates was studied for 10 years, while the trends of incidence were determined by the least squares method:

**y= a + bx**                    ** (3)**

where: **y** – equalized rate;

**x** – a conditional series of numbers arranged symmetrically with respect to zero;

**a** – conditional average;

**b** – alignment factor.

To calculate the average annual growth rate and/or growth rate of the dynamic series, the geometric mean equal to the root of the power of n from the product of the annual growth rate indicators was used:

$T_{\mathrm{up}/\mathrm{down}}=\sqrt[{2}]{T_{1}\times T_{2}\times T_{3}\times \ldots T_{n}}$                     (4)

where **T** – annual growth and/or upward/downward rates

**n** – the number of indicators.

When compiling cartograms, crude rates and ASRs were used for 10 years (2009-2018). The method of compiling a cartogram proposed in 1974 by S.I. Igisinov was used, based on the determination of the standard deviation (σ) from the average (x). The scale of steps was calculated as follows: taking σ as an interval, the maximum and minimum levels of incidence were determined according to the formula: x±1.5 σ, with the minimum indicator equal to x–1.5 σ and the maximum equal to x+1.5 σ. After that, we determined the scale of the cartogram steps: 1) (x–1,5σ)+σ; 2) (x–1,5σ)+2σ; 3) (x-1.5 σ)+3σ, etc., and the indicators were grouped according to the formula x±0.5 σ, corresponding to the average level (x-0.5 σ and x+0.5 σ), and the values that are separated from the average level of incidence by σ show reduced ((x–0.5 σ)–σ) and increased ((x–0.5 σ)–σ) incidence rate.

Viewing and processing of the received materials was carried out using the Microsoft 365 software package (Excel, Word, PowerPoint), in addition, online statistical calculators were used (https://medstatistic.ru/calculators/averagestudent.html), where Student criterion was calculated when comparing the average values.

*Ethics approval*

Because this study involved the analysis of publicly available administrative data and did not involve contacting individuals, consideration and approval by an ethics review board was not required. At the same time, the submitted data is in accordance with the Law of the Republic of Kazakhstan No. 257-IV of March 19, 2010 “About State statistics” (http://adilet.zan.kz/rus/docs/Z100000257), the information in the summary report is confidential and can only be used for statistical purposes in accordance with the Principles of the World Medical Association (WMA, 2013).

**Figure 1:**
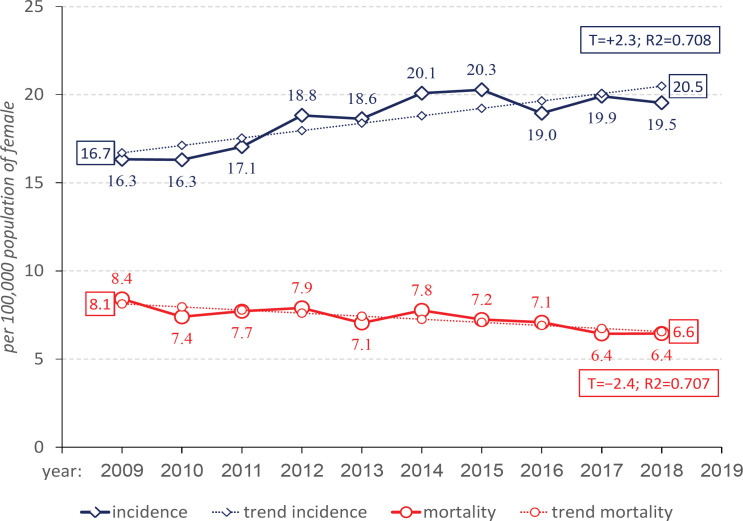
Dynamics of Crude Incidence and Mortality Rates of Cervical Cancer in Kazakhstan, 2009-2018, per 100,000 population of female

**Figure 2:**
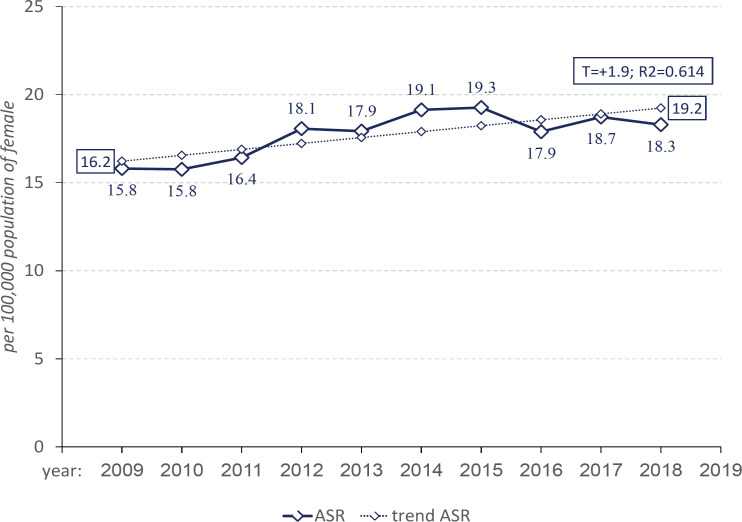
Dynamics of Age-Standardized Incidence Rate of Cervical Cancer in Kazakhstan, 2009-2018, per 100,000 population of female

**Table 1:** Number of Cervical Cancer and Average Age of Patients by Regions of Kazakhstan, 2009-2018

Regions	Number (%)*	Average age, years		Т_up/down_, %	R^2^
		P±m	95% CI		
Mangistau	389 (2.4)	50.1±0.5	49.1-51.0	+0.4	0.152
Kyzylorda	476 (2.9)	51.5±0.7	50.1-53.0	+0.4	0.078
North Kazakhstan	508 (3.1)	51.1±0.7	49.8-52.5	+0.6	0.238
Atyrau	592 (3.6)	49.0±0.7	47.7-50.4	+0.9	0.415
Astana city	640 (3.9)	49.5±0.5	48.5-50.4	+0.2	0.039
West Kazakhstan	682 (4.1)	49.8±0.7	48.4-51.1	+0.7	0.271
Zhambyl	765 (4.7)	52.2±0.4	51.3-53.0	+0.6	0.510
Akmola	790 (4.8)	51.3±0.5	50.4-52.2	+0.3	0.115
Aktobe	851 (5.2)	51.0±0.4	50.2-51.8	±0.0	0.001
Pavlodar	899 (5.5)	51.1±0.6	50.0-52.2	+0.2	0.041
Kostanay	1119 (6.8)	48.3±0.5	47.4-49.3	+0.1	0.006
Karaganda	1290 (7.8)	52.1±0.3	51.5-52.7	+0.2	0.197
South Kazakhstan	1688 (10.3)	50.7±0.5	49.7-51.7	+0.8	0.604
East Kazakhstan	1716 (10.4)	50.8±0.6	49.6-52.0	+0.8	0.455
Almaty	1996 (12.1)	51.9±0.3	51.3-52.4	+0.1	0.028
Almaty city	2040 (12.4)	49.9±0.6	48.7-51.1	−0.7	0.297
Kazakhstan	16441 (100.0)	50.7±0.2	50.3-51.0	+0.3	0.579

* the table is built taking into account the sorting from A to Z of the number of patients

**Figure 3:**
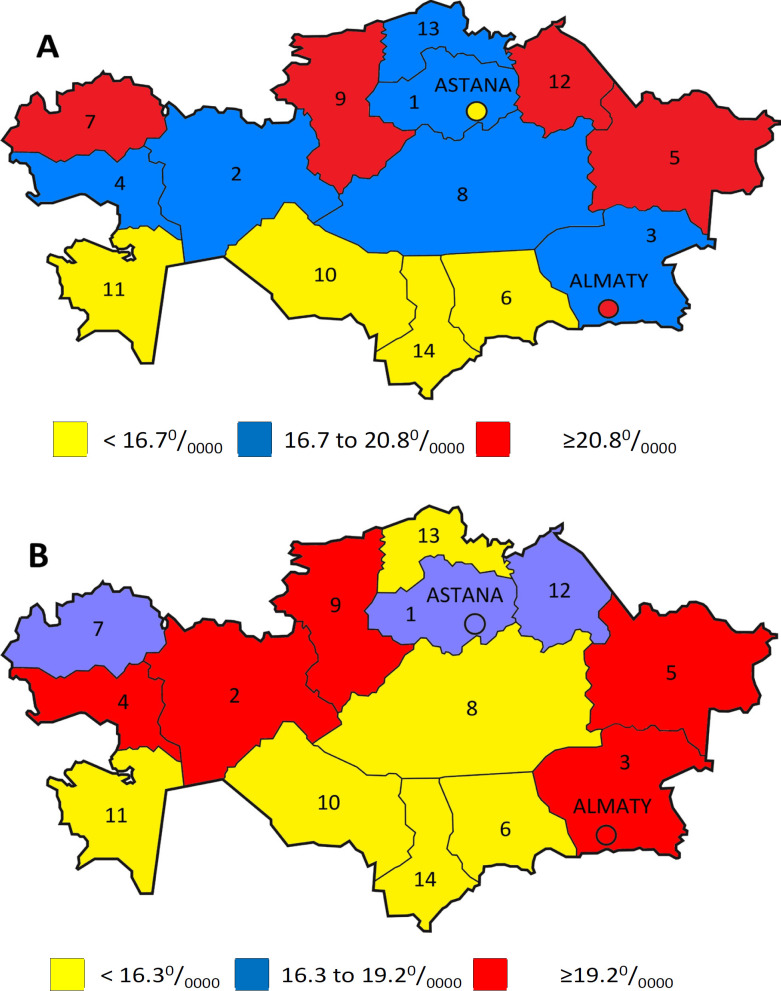
Cartogram of cervical cancer incidence in Kazakhstan, 2009-2018 (A – CR; B – ASR). Regions: 1. Akmola, 2. Aktobe, 3. Almaty, 4. Atyrau,5. East-Kazakhstan, 6. Zhambyl, 7. West-Kazakhstan,8. Karaganda, 9. Kostanay, 10. Kyzylorda,11. Mangystau, 12. Pavlodar, 13. North-Kazakhstan, 14. South-Kazakhstan

**Table 2:** The Average Age-Specific Incidence Rates of Cervical Cancer in Kazakhstan for the Years 2009-2018

Age groups, years	ASIR, per 100,000 population of female		Т_up/down_, %	R^2^
	M±m	95% CI		
<30	1.3 ± 0.1	1.1-1.4	−1.8	0.111
30-34	17.0 ± 0.5	15.9-18.0	+1.7	0.306
35-39	29.7 ± 0.9	28.0-31.4	−0.9	0.103
40-44	42.0 ± 1.6	38.8-45.1	+2.9	0.552
45-49	43.8 ± 1.9	40.1-47.4	+4.1	0.878
50-54	45.3 ± 1.1	43.1-47.5	+2.0	0.650
55-59	43.2 ± 1.7	39.8-46.6	+1.9	0.219
60-64	40.5 ± 0.9	38.8-42.2	+1.4	0.402
65-69	37.4 ± 2.2	33.1-41.7	+4.4	0.537
70-74	30.2 ± 1.0	28.3-32.0	+0.7	0.044
75-79	28.4 ± 1.9	24.7-32.1	+0.6	0.009
80-84	22.2 ± 2.3	17.7-26.8	+2.3	0.047
≥85	15.0 ± 1.9	11.2-18.7	+0.02	0.000

**Table 3:** Average ASIR of Cervical Cancer in the Regional Context, 2009-2018

Regions	Indicators	Age groups, years					
		<30	30-39	40-49	50-59	60-69	≥70
Akmola	P±m	1.2±0.2	26.2±1.9	42.8±3.4	42.3±2.5	37.4±3.8	25.0±4.8
	Т_up/down_, %	−8.9	+0.4	−0.5	+1.8	+0.5	−1.8
	R^2^	0.179	0.002	0.040	0.091	0.002	0.009
Aktobe	P±m	1.1±0.3	24.4±2.2	45.7±5.1	50.3±3.9	50.7±5.1	32.2±2.8
	Т_up/down_, %,	+10.2	+3.5	+10.6	+5.6	+4.4	+1.7
	R^2^	0.141	0.151	0.733	0.479	0.186	0.037
Almaty	P±m	0.9±0.1	21.6±2.8	43.1±5.1	60.5±3.8	50.4±3.2	32.8±3.3
	Т, %	−4.0	+1.1	+3.0	+0.5	+0.7	−0.4
	R^2^	0.143	0.007	0.065	0.006	0.012	0.001
Atyrau	P±m	1.1±0.2	27.1±3.5	56.9±2.0	55.7±6.0	49.8±4.2	22.9±4.4
	Т_up/down_, %,	−14.0	−2.5	+0.9	+2.9	+0.8	+14.4
	R^2^	0.487	0.037	0.064	0.072	0.008	0.402
East Kazakhstan	P±m	2.0±0.3	28.7±2.7	53.0±4.4	45.9±2.2	32.9±3.2	25.2±1.7
	Т_up/down_, %,	−5.7	−5.9	+2.7	+2.6	+4.8	−2.0
	R^2^	0.197	0.393	0.099	0.300	0.229	0.089
Zhamby	P±m	0.6±0.1	16.0±1.4	29.0±2.0	41.7±3.7	40.5±2.3	27.2±3.1
	Т_up/down_, %,	+8.1	−3.6	+0.1	−1.3	−0.7	+6.9
	R^2^	0.109	0.163	0.000	0.023	0.017	0.319
West Kazakhstan	P±m	0.8±0.2	29.4±3.5	53.8±4.5	45.3±3.0	28.8±6.1	22.3±2.5
	Т_up/down_, %,	+0.7	+2.0	+2.0	+3.3	+14.4	+7.1
	R^2^	0.000	0.027	0.056	0.235	0.332	0.357
Karaganda	P±m	1.5±0.3	21.2±1.5	35.3±1.9	34.6±1.9	34.3±2.2	27.3±1.5
	Т_up/down_, %,	−11.2	+0.4	+4.7	+2.0	−2.4	+0.6
	R^2^	0.250	0.002	0.698	0.135	0.141	0.013
Kyzylorda	P±m	0.8±0.3	13.3±1.9	31.8±3.4	35.8±3.2	42.3±4.1	33.3±3.1
	Т_up/down_, %,	−0.5	+0.3	−0.5	+1.2	−5.6	+1.8
	R^2^	0.000	0.000	0.002	0.018	0.340	0.039
Kostanay	P±m	3.9±0.4	40.1±3.2	52.7±4.8	35.5±2.6	33.0±2.8	21.0±2.3
	Т_up/down_, %,	+4.1	−1.1	+8.5	+0.9	+4.3	−4.1
	R^2^	0.126	0.021	0.736	0.017	0.251	0.141
Mangistau	P±m	0.4±0.1	13.3±1.7	39.9±2.7	48.5±2.6	29.6±2.9	22.1±5.9
	Т_up/down_, %,	+1.4	+4.5	+2.6	+2.9	+5.9	+13.7
	R^2^	0.001	0.122	0.148	0.298	0.329	0.193
Pavlodar	P±m	1.3±0.3	24.3±2.3	49.4±4.8	48.9±4.6	32.2±3.9	26.0±2.4
	Т_up/down_, %,	−6.6	−0.7	+5.8	+6.1	−1.0	−1.1
	R^2^	0.112	0.006	0.323	0.389	0.007	0.015
North Kazakhstan	P±m	1.7±0.4	22.0±3.4	35.7±4.3	23.1±2.2	32.4±4.4	16.9±2.9
	Т_up/down_, %,	+2.7	+0.5	+4.5	+0.3	+7.9	+1.1
	R^2^	0.017	0.001	0.132	0.000	0.291	0.004
South Kazakhstan	P±m	0.4±0.1	13.6±0.9	37.0±2.9	43.6±2.4	37.8±4.4	20.8±2.2
	Т_up/down_, %,	−21.5	−1.4	+4.2	+2.3	+9.8	+4.0
	R^2^	0.678	0.047	0.277	0.176	0.593	0.135
Almaty city	P±m	2.9±0.3	32.8±3.3	48.6±2.7	49.6±4.2	50.9±4.0	32.8±3.2
	Т, %	+4.7	+4.6	+3.5	+1.1	+1.0	−3.8
	R^2^	0.241	0.198	0.384	0.016	0.016	0.153
Astana city	P±m	1.1±0.2	19.3±1.4	37.3±2.5	38.2±3.3	37.7±5.0	43.7±4.4
	Т_up/down_, %,	−4.6	−4.3	−0.3	−3.8	+2.6	−5.6
	R^2^	0.041	0.377	0.002	0.193	0.038	0.325
Kazakhstan	P±m	1.3±0.1	23.0±0.6	42.8±1.6	44.3±1.2	39.2±1.2	21.6±0.7
	Т_up/down_, %,	−1.8	−0.1	+3.5	+1.9	+2.5	+0.2
	R^2^	0.111	0.001	0.851	0.504	0.699	0.004

**Table 4:** Changes in the Age-Standardized Incidence Rate of Cervical Cancer in 2009-2018

Regions	ASR, per 100,000 population of female			Significance		Т_up/down_, %*	R^2^
	2009	2018	Mean	t	p		
Astana city	21.5±2.8	17.5±1.9	16.4±0.8	1.16	0.25	−2.0	0.179
Zhambyl	15.2±1.8	14.2±1.6	14.5±0.5	0.00	1.00	−0.3	0.011
Kyzylorda	13.6±2.2	15.7±2.1	14.4±0.7	1.26	0.21	−0.3	0.002
Akmola	16.9±2.0	19.1±2.1	17.7±0.4	0.90	0.37	+0.3	0.019
East Kazakhstan	17.7±1.5	18.9±1.5	19.8±1.0	1.37	0.17	+0.3	0.003
Karaganda	12.2±1.2	15.5±1.4	15.2±0.5	1.98	0.05	+1.2	0.144
Almaty	18.7±1.4	17.5±1.3	20.1±1.5	0.10	0.92	+1.2	0.024
Atyrau	21.6±3.1	25.6±3.0	21.7±1.2	1.11	0.27	+1.2	0.052
Kazakhstan	15.8±0.4	18.3±0.4	17.7±0.4	4.42	0.00	+1.9	0.613
Almaty city	18.1±1.5	17.7±1.3	22.0±1.2	0.05	0.96	+2.2	0.166
Pavlodar	15.3±1.9	21.3±2.1	18.7±1.0	2.57	0.01	+2.9	0.311
Kostanay	17.8±1.9	26.3±2.3	20.9±1.1	3.02	0.00	+2.9	0.324
North Kazakhstan	13.5±2.0	14.1±2.1	13.9±1.2	0.45	0.65	+3.0	0.127
South Kazakhstan	13.0±1.1	13.7±1.0	14.8±0.7	0.39	0.70	+3.5	0.467
West Kazakhstan	15.2±2.2	25.8±2.7	19.0±1.2	3.08	0.00	+3.8	0.368
Mangistau	12.2±2.5	21.1±2.8	15.2±0.9	2.41	0.02	+4.1	0.482
Aktobe	15.0±2.0	27.7±2.5	20.0±1.3	4.47	0.00	+6.3	0.846

*The Table is built taking into account the sorting from A to Z of the Т_up/down_ value

## Discussion

In 2009-2018, 16,441 new CC cases were registered in the Republic of Kazakhstan. The average age of patients in Kazakhstan 50.7±0.2 year. Regions with a lower average age of patients are Kostanay (48.3±0.5) and high average age is in Zhambyl (52.2±0.4). The average age trends show that they are growing in almost all regions: from T_up_=+0.1% (Almaty) to T_up_=+0.9% (Atyrau) (Table 1).

The crude rate of CC incidence in the republic was 18.6±0.5 per 100,000 population of female, and it grew from 16.3±0.4 in 2009 to 19.5±0.5 in 2018 (Figure 1) in dynamics, the difference was statistically significant (p=0.000). Crude rates of mortality declined from 8.4±0.3 in 2009 to 6.4±0.3 in 2018 (p=0.000, Figure 1) and averaged equaled to 7.3±0.2 per 100,000 population of female. In the dynamics, the aligned crude rate repeated the revealed trend towards an increase in T_up_=+2.3% (incidence) and decrease − T_down_= −2.4% (mortality).

The ASR in Kazakhstan was equal to 17.7±0.4 cases per 100,000 population of female. Over time, the leveled ASR were growing (p=0.000; R^2^=0.615), with the average annual upward rate of T_up_=+1.9% (Figure 2).

CC occurrence and incidence were directly related to the age composition of the population since the age was one of the most important risk factors. Thus, the analysis of ASIR has shown a high in the age groups of 45-64 years per 100,000 population of female: 43.8 in 45-49 years, 45.3 in 50-54 years, 43.2 in 55-59 years, and 40.5 in 60-64 years (Table 2).

CC incidence had a downward trend only in two studied age groups: below 30 years (T_down_=−1.8%) and in the age of 35-39 years (T_down_=−0.1%). In other age groups, the leveled CC incidence was growing, with the most pronounced annual average upward rates in the age groups of 40-44 (T_up_=+2.9%), 45-49 (T_up_=+4.1%), and 65-69 years (T_up_=+4.4%) (Table 2).

The analysis of ASIR in the context of regions showed (Table 3) that mainly they had a unimodal peak of morbidity. So, the peak of incidence per 100,000 population of female at age 40-49 was in Karaganda (35.3), Akmola (42.8), Pavlodar (49.4), Kostanay (52.7), East-Kazakhstan (53.0), West-Kazakhstan (53.8) and Atyrau (56.9) regions. The peak incidence per 100,000 population of female at age 50-59 was established in Zhambyl (41.7), South-Kazakhstan (43.6), Mangystau (48.5) and Almaty (60.5) regions. The peak incidence per 100,000 population of female at age 60-69 was in Kyzylorda (42.3), Aktobe (50.7) regions, and Almaty city (50.9). The peak incidence at 70 years and older was detected only in Astana city (43.7 per 100,000 population of female). In North-Kazakhstan region bimodal growth was established with peaks at 40-49 years (35.7 per 100,000 population of female) and 60-69 years (32.4 per 100,000 population of female).

The trends of ASIR according to regions had a different tendency. Thus, in the age group under 30, the most pronounced values of the average annual growth rate with approximation data was established in South-Kazakhstan (T_down_=−21.5; R^2^=0.678) region. High ASIR decline in the age group 30-39 were revealed in East-Kazakhstan region (T_down_=−5.9; R^2^=0.393) and growth in Almaty city (T_up_=+4.6; R^2^=0.198). In 40-49 years, a very insignificant decrease was noted only in the Akmola region (T_down_=−0.5; R^2^=0.040) and Astana city (T_down_=−0.3; R^2^=0.002), in other regions there was a tendency of growth and most pronounced in Aktobe (T_up_=+10.6; R^2^=0.733), Karaganda (T_up_=+4.7; R^2^=0.698) and Kostanay (T_up_=+8.5; R2=0.736) regions (Table 3). The decrease of ASIR at age group 50-59 was found in Zhambyl region (T_down_=−1.3; R^2^=0.023) and Astana city (T_down_=−3.8; R^2^=0.193), in other regions there was an upward trend, which was the most pronounced in Aktobe (T_up_=+5.6; R^2^=0.479) and Pavlodar (T_up_=+6.1; R^2^=0.389) regions. The incidence rates at 60-69 years had a different tendency, with a pronounced downward trends revealed in Kyzylorda region (T_down_=−5.6; R^2^=0.340), and upward trends in South-Kazakhstan region (T_up_=+9.8; R^2^=0.593). The incidence trends at age group 70 years and older were the most pronounced in Astana city (T_down_=−3.8; R^2^=0.325) and Atyrau region (T_up_=+14.4; R^2^=0.402) (Table 3).

In dynamics, ASR tended to decrease in two regions: Zhambyl (T_down_=−0.3%; R^2^=0.011) and Kyzylorda (T_down_=−0.3%; R^2^=0.002), as well as in the city of Astana (T_down_=−2.0%; R^2^=0.179). However, the changes were not statistically significant, as indicated by the low values of the approximation (Table 4).

In other regions, trends in ASR were growing, while a statistically significant difference between the indicators of 2009 and 2018 was found in the following areas: Aktobe (p=0.00), Kostanay (p=0.00), West Kazakhstan (p=0.00), Pavlodar (p=0.01), Mangistau (p=0.02), Karaganda (p=0.05) and the Republic of Kazakhstan (p=0.00) (Table 4).

Based on the calculated average annual CR and ASR CC indicators, the cartograms were compiled. The levels of CC CR per 100,000 population of female based on the following criteria were determined: low – up to 16.7, average – from 16.7 to 20.8, high – above 20.8. As a result, the following groups of regions were revealed (Figure 3A):

Regions with the lowest indicators (up to 16.7 per 100,000 population of female): South Kazakhstan (12.3), Kyzylorda (13.0), Mangystau (13.3), Zhambyl (13.9) and Astana city (15.6).

Regions with average indicators (from 16.7 to 20.8 per 100,000 population of female): North Kazakhstan (16.8), Karaganda (17.9), Aktobe (20.3), Almaty (20.4), Atyrau (20.6) and Akmola (20.76).

Regions with high indicators (19.2 and above per 100,000 population of female): West Kazakhstan (21.2), Pavlodar (22.6), East Kazakhstan (23.5), Kostanay (24.1) and Almaty city (24.1).

The levels of CC ASR per 100,000 population of female based on the following criteria were determined: low – up to 16.3, average – from 16.3 to 19.2, high – above 19.2. As a result, the following groups of regions were determined (Figure 3B):

Regions with the lowest indicators (up to 16.3 per 100,000 population of female): North Kazakhstan (13.9), Kyzylorda (14.4), Zhambyl (14.5), South Kazakhstan (14.8), Mangystau (15.2) and Karaganda (15.2).

Regions with average indicators (from 16.3 to 19.2 per 100,000 population of female): Astana city (16.4), Akmola (17.7), Pavlodar (18.7) and West Kazakhstan (19.0).

Regions with high indicators (19.2 per 100,000 population of female and above): East Kazakhstan (19.8), Aktobe (20.0), Almaty (20.1), Kostanay (20.9), Atyrau (21.7), Almaty city (22.0).

Thus, the incidence cartograms more clearly reflect the spatial distribution of CC in the republic, while the discrepancy between the theoretical and actual distribution of CC incidence by regions and cities is small, the Pearson criterion (χ2) equals 9.0 and 7.8 (for a crude and age-standardized indicator, respectively).

## Discussion

The growth of CC incidence in Kazakhstan corresponds to the global trend. However, the incidence is the lowest in the developed countries, while less developed countries account for more than 85% of the global CC burden (Randall and Ghebre, 2016; Chuang et al., 2016).

The average age of patients with CC in the republic during the study period was 50.7 years and this indicator decreased by 2.8 years compared to our previous data (Igissinov et al., 2012).

Age analysis of CC incidence in Kazakhstan has revealed a unimodal growth with a peak of incidence at the age of 50-54 years (44.3 cases per 100,000 population of female). The same pattern is observed in Hungary, Russia, India, and Ukraine. The average age is close to Germany (50 years in Kazakhstan vs. 52 years in Germany) (Bray et al, 2018). 

The analysis of age-specific features of CC incidence according to our data showed that in the age group under 30, the lowest incidence rate was in Mangystau region (0.4 cases per 100,000 population of female), the highest rate in Kostanay region (3.9 cases per 100,000 population of female). According to IARC (Ferlay et al., 2020), the lowest rates in this age group were found in Saudi Arabia (0.04 cases per 100,000 population of female) and the highest in Mozambique (8.1 cases per 100,000 population of female). In 30-39, low rate was also found in Mangystau region (13.3 cases per 100,000 population of female) and the highest – in Kostanay region (40.1 cases per 100,000 population of female). The lowest rate in this age group was found in Iraq (0.99 cases per 100,000 population of female) and the highest was in Eswatini (90.7 cases per 100,000 population of female). In 40-49, the lowest rate – in Zhambyl region (29.0 cases per 100,000 population of female) and the highest in Atyrau region (56.9 cases per 100,000 population of female). The lowest rate was in the Gaza Strip and West Bank (1.0 cases per 100,000 population of female) and the highest in Eswatini (185.0 cases per 100,000 population of female). In 50-59 years, low rate was in North-Kazakhstan region (23.1 cases per 100,000 population of female), and the maximum – in Almaty region (60.5 cases per 100,000 population of female). According to IARC, the lowest rate was in Luxembourg (4.6 cases per 100,000 population of female), while the highest also in Eswatini (287.2 cases per 100,000 population of female). In 60-69 years old, low rate was in West-Kazakhstan region (28.8 cases per 100,000 population of female), and the maximum in Almaty city (50.9 cases per 100,000 population of female). The lowest rate was in Iceland (5.6 cases per 100,000 population of female) and the highest in Tanzania (298.8 cases per 100,000 population of female). In the 70+ age group, low rate was found in North-Kazakhstan region (16.9 cases per 100,000 population of female), and the highest rate in Astana city (43.7 cases per 100,000 population of female), the lowest rate was found in Finland (6.60 cases per 100,000 population of female) and the highest in the Republic of the Gambia (360.2 cases per 100,000 population of female).

The spatial analysis of CC incidence indicates geographical variability, so low incidence rates (per 100,000 population of female) were established in the southern regions of the republic: South Kazakhstan (12.3), Kyzylorda (13.0), Mangystau (13.3) and Zhambyl (13.9), this trend has continued since the last study was performed (Igissinov et al., 2012), but the indicator values have increased. It should be noted that there have also been changes in the distribution of areas in the regions of medium and high incidence. Thus, Almaty, Karaganda, Atyrau, and Akmola regions with a high level of incidence have moved to regions with an average level of incidence. And the East Kazakhstan, which belonged to the regions with an average level of incidence, moved to a high level (Igissinov et al., 2012). In our opinion, the geographical variability of the CC incidence is associated with demographic factors (ethnic composition of the population, changes in the age structure, migration) and risk factors for getting sick (Telmanova et al., 2020), besides, they are associated with the level of medical care, with the screening performing, which in general led to a decrease in mortality in the republic. A more detailed study is planned at the regional level in the future.

An overall increase in incidence is registered in almost all groups of studied female population, with the most pronounced trend at the age of 45-49 years (R^2^=0.878) and 65-69 years (R^2^=0.537). The same pattern is observed in Shanghai (China) (Huang et al., 2016) and Germany, although some studies report a decrease in incidence.

We associate the factors contributing to the decrease or increase of incidence with the organization of CC accounting and registration, as well as with anti-cancer activities conducted in the Republic, including cytological screening for CC, which has become a part of mass preventive examinations since 2008.

CC is often associated with a pre-cancerous lesion such as Cervical Intraepithelial Neoplasia, adequate treatment includes: detection of an infectious agent in women, conservative etiotropicand pathogenetic treatment aimed at complete disappearance or reduction of virulent effect of infection in 2 stages with application of general and local effects. The criterion for the effectiveness of the treatment is the result of an objective examination (examination of the neck in mirrors, oncocytology) and an immunoenzyme blood test for STI infections, in the presence of viruses - a decrease in titer readings to an acceptable indicator in which the infection does not have a virulent property. Therefore, CC screening and adequate treatment of cervical pathologies, including dysplasia, can prevent the development of CC. Therefore, the cytological screening of cervix can prevent the development of CC. In Korean studies, CC screening using a PAP test has reduced CC incidence by 62% in the cohort study and by 65% in the case-control group study, as well as the risk of mortality from CC by 64%. The combination of the HPV test with cervical cytology has further reduced the CC incidence compared to only cervical cytology (Min et al., 2015).

A decrease in mortality among CC patients is mainly associated with systemic and opportunistic screening programs, such as routine PAP tests 16, which can be used to detect changes in cytology (Weiderpass and Labrèche, 2012). The implementation of a screening program in Kazakhstan shows good results. In 2007, every 4th woman had an advanced stage at diagnosis; that is, the neglect rate was 26.7%. Since 2008, with the introduction of the state СС screening program, the neglect rate has fallen by half. Thus, we identified in our previous research of the study period reveals a trend: early diagnosis indicators (specific weight of patients with I-II stage) improved from 79.8% (2009) to 88.1% in 2018, and accordingly the specific weight of neglected patients significantly decreased with stage III (from 15.4% to 8.9%) and with stage IV (from 3.4% to 2.7%). The morphological verification indicators for CC remained virtually unchanged, remaining fairly high 99.2% and 99.3%, respectively, in 2009 and 2018. (Igissinov et al., 2020)

Organized programs with systematic appeals, feedback, as well as control and monitoring systems have shown the most excellent effect (e.g., in Finland and Iceland). At the same time, they require fewer resources than unorganized programs (like in the US or Russia). Many screening programs conducted in developing countries utilize an unrealistic concept of frequently repeated screening tests (e.g., annual) and are aimed at women of a wide age range (20-65 years). However, it would be more efficient to test high-risk women (e.g., those aged 35-49 or 30-50) only once or twice with good quality and a highly sensitive test, with an emphasis on a broad coverage (>80%) of the target female audience (Sankaranarayanan et al., 2001).

Developed countries use PAP test+HPV test for screening purposes (Konnon and Soyunov, 2018), while Kazakhstan is using only the PAP test. For that reason, the quality of screening in our country is lower than in more developed countries. Prolonged exposure to HPV and other various urogenital infections results in immunodeficient conditions in women, which induce the risk of pathological conditions of the cervical mucosa, including CC. With weakening of immunity, in concomitant inflammatory diseases of the genitals, activation and development of abnormalities in the cervical epithelium, that is, dysplasia, is possible (zur Hausen and de Villiers, 1994; Fernandes et al., 2015; Song et al., 2015).

Limitations of the current study include the quality of the primary data. Since there are changes in the policy of providing cancer care, and accounting and registration require additional resources, this may eventually affect the results of the study. Currently, it is not yet possible to get data from the reporting forms for ethnic groups, not in the forms of the district level (we have data for regions, which in its turn consist of districts). Such data would allow us to study more deeply the issues of epidemiology, in particular ethnoepidemiology, and to make district cartograms. Thus, at this moment we have generalized data. Nevertheless, there is no doubt about it, since the results of the analysis show that the common trends in the incidence of cervical cancer in this study coincide with the same data in the world. Consequently, our findings should be correct.

Thus, the study of CC incidence trends is of both theoretical and practical interest and plays an essential role in monitoring and assessment of screening programs implemented in the country and secondary prevention of cervical pathology. Health authorities should consider the obtained results in the arrangement of anti-cancer activities.

## Author Contribution Statement

ZK, AA, ZhB, DiT, AM, MZ, AK, VO – Collection and preparation of data, primary processing of the material and their verification. ZaB, DK, ZT, SK, YK – Statistical processing and analysis of the material, writing the text of the article (material and methods, results). GI, SU, DuT, GN, AO – Writing the text of the article (introduction, discussion). NI, GI, ZT – Concept, design and control of the research, approval of the final version of the article.

All authors approved the final version of the manuscript.
